# Role of systemic inflammation scores for prediction of clinical outcomes in patients treated with atazanavir not boosted by ritonavir in the Italian MASTER cohort

**DOI:** 10.1186/s12879-017-2322-z

**Published:** 2017-03-15

**Authors:** Maria Concetta Postorino, Mattia Prosperi, Emanuele Focà, Eugenia Quiros-Roldan, Elisa Di Filippo, Franco Maggiolo, Alberto Borghetti, Nicoletta Ladisa, Massimo Di Pietro, Andrea Gori, Laura Sighinolfi, Angelo Pan, Nicola Mazzini, Carlo Torti

**Affiliations:** 1 Infectious and Tropical Diseases Unit, Department of Medical and Surgical Sciences, University “Magna Graecia” of Catanzaro, Catanzaro, Italy; 2 Department of Epidemiology, College of Public Health and Health Professions & College of Medicine, University of Florida, Gainesville, USA; 3 University Department of Infectious and Tropical Diseases, University of Brescia and Spedali Civili General Hospital, Brescia, Italy; 4 Clinic of Infectious Diseases of “Papa Giovanni XXIII” Hospital of Bergamo, Bergamo, Italy; 5 Institute of Clinical Infectious Diseases of Catholic University of Sacred Heart, Rome, Italy; 6 Clinic of Infectious Diseases, University of Bari, Bari, Italy; 7 Clinic of Infectious Diseases of “Azienda Ospedaliera S.M. Annunziata”, Florence, Italy; 8 Clinic of Infectious Diseases, San Gerardo de’ Tintori Hospital, Monza, Italy; 9 Clinic of Infectious Diseases of “Azienda Ospedaliera S. Anna” of Ferrara, Ferrara, Italy; 10 Clinic of Infectious Diseases of “Istituti Ospitalieri” of Cremona, Cremona, Italy; 11 MISI Foundation, Brescia, Italy

**Keywords:** HIV, Atazanavir, Ritonavir, Clinical events, Systemic inflammation scores

## Abstract

**Background:**

Atazanavir (ATV) not boosted by ritonavir (uATV) has been frequently used in the past for switching combination antiretroviral therapy (cART). However, the clinical outcomes and predictors of such strategy are unknown.

**Methods:**

An observational study was carried out on the Italian MASTER, selecting HIV infected patients on cART switching to an uATV-containing regimen. Baseline was set as the last visit before uATV initiation. In the primary analysis, a composite clinical end-point was defined as the first occurring of any condition among: liver, cardiovascular, kidney, diabetes, non AIDS related cancer or death events. Incidence of AIDS events and incidence of composite clinical end-point were estimated. Kaplan-Meier and multivariable Cox regression analysis were used to assess predictors of the composite clinical end-point.

**Results:**

436 patients were observed. The majority of patients were males (61.5%) and Italians (85.3%), mean age was 42.7 years (IQR: 37.7–42), the most frequent route of transmission was heterosexual intercourse (47%), followed by injection drug use (25%) and homosexual contact (24%); the rate of HCV-Ab positivity was 16.3%. Patients were observed for a median time of 882 days (IQR: 252-1,769) under uATV. We recorded 93 clinical events (3 cardiovascular events, 20 kidney diseases, 33 liver diseases, 9 non AIDS related cancers, 21 diabetes, 7 AIDS events), and 19 deaths, accounting for an incidence of 3.7 (composite) events per 100 PYFU. At multivariable analysis, factors associated with the composite clinical end-point were intravenous drug use as risk factor for HIV acquisition *vs.* heterosexual intercourses [HR: 2.608, 95% CI 1.31–5.19, *p* = 0.0063], HIV RNA per Log_10_ copies/ml higher [HR: 1.612, 95% CI 1.278–2.034, *p* < 0.0001], number of switches in the nucleoside/nucleotide (NRTI) backbone of cART (performed to compose the uATV regimen under study or occurred in the past) per each more [HR: 1.085, 95% CI 1.025–1.15, *p* = 0.0051], Fib-4 score per unit higher [HR: 1.03, 95% CI 1.018–1.043, *p* < 0.0001] and Neutrophil/lymphocytes ratio (NLR inflammation score) per Log_10_ higher [HR: 1.319, 95% CI 1.047–1.662, *p* = 0.0188].

**Conclusions:**

Intravenous drug users with high HIV RNA, high Fib-4 levels and more heavily exposed to antiretroviral drugs appeared to be more at risk of clinical events. Interestingly, high levels of inflammation measured through NLR, were also associated with clinical events. So, these patients should be monitored more strictly.

## Background

Atazanavir (ATV) is a protease inhibitor (PI) for the treatment of HIV infection, prescribed with ritonavir (atazanavir/ritonavir, ATV/r) in combination antiretroviral therapy (cART) [1–4]. Differently from other PIs, ATV not boosted by ritonavir (unboosted ATV, uATV) has been rather frequently used in the past as an off-label switching option [5, 6], for toxicity reasons [7, 8]. Previous studies indicated safety and effectiveness of uATV prescription in selected cART experienced patients, with undetectable HIV RNA and without drug resistances [9]. This is why regimens including uATV have been experimented with and were used in clinical practice [6], and subsequently uATV prescription has been formally approved in the meantime by European Medicine Agency (EMA) and by Italian National Health authorities in 2014. At the same time, national guidelines recommended caution because of the lower genetic barrier of regimens including uATV [10].

Clinical outcome of patients receiving ATV (and particularly of those received uATV) is still not completely clear. In a previous study of the Italian MASTER cohort [11], patients treated with ATV/r more frequently achieved a composite outcome of success (HIV RNA <500 copies/mL, no AIDS events, CD4 + T cell count >500 cell/mm^3^) than patients prescribed other PIs [12] and reported a lower probability of switching the regimen than patients treated with other PIs [12].

However, there are some unknowns about cardiovascular risk and renal and liver profile of patients treated with uATV. Firstly, a moderate increase of lipid values (total cholesterol, HDL and LDL fractions, triglycerides) has been reported in patients prescribed ATV/r [13, 14]. Similarly, the expected increase in cardiovascular risk [15] was not confirmed by previous studies [16–18]. A recent study of the Italian MASTER cohort reported that the increased total cholesterol was balanced by the increased HDL fraction in patients prescribed ATV/r or uATV [19] and other studies observed a less impact on lipid profile in patients prescribed uATV [20].

Some degrees of acute and chronic interstitial nephritis, crystal deposition and nephrolithiasis were described when ATV/r was co-administered with other potentially nephrotoxic drugs, as tenofovir (TDF) [21–23]. However, an improvement of filtration rate in patients prescribed ATV/r or uATV was reported, either after switching to a regimen without TDF [19] or in patients prescribed ATV/r as a part of simplification regimens [13, 14]. Therefore, other factors than use of ATV may be involved in renal failure development.

Liver toxicity is a well-known side effect of ATV/r, so prescription of uATV is often adopted in case of severe hyperbilirubinaemia and cholelitiasis [7]. However, effects of uATV prescription on liver fibrosis progression are not yet known.

Further, an increased risk of clinical events was associated with systemic inflammation levels in HIV infected patients. As recently described, neutrophil/lymphocyte ratio (NLR) measured at baseline and during follow up was independently associated with incidence of cardiovascular events [24], and both NLR and platelet/lymphocyte ratio (PLR) have been found to be associated with risk of death in patients with solid cancer, in general population and HIV positive subjects [25].

Lastly, among proposed serum non-invasive biomarkers of liver fibrosis, the Fib-4 score has been validated both in HIV/HCV co-infected and in HIV mono-infected patients and it was a reliable driver of both liver fibrosis progression and clinical events [26, 27].

The goals of present study were: (i) to estimate the risk of clinical progression (including both AIDS and non AIDS related morbidities, and death) in patients prescribed uATV; (ii) to explore possible predictors of the aforementioned outcomes, including systemic inflammation scores.

## Methods

### Characteristics of the Italian MASTER cohort

The present study was conducted including patients enrolled in the Italian MASTER cohort (*MAnagement Standardizzato di TErapia antiRetrovirale*, Standardized Management of Antiretroviral Therapy), a longitudinal multicenter cohort including patients in nine referral centers throughout Italy (http://www.mastercohort.it) [11]. Patients’ data are recorded on a common electronic clinical chart software (Health & Notes 3.5W, Healthware S.p.A., Naples, Italy) employed by all participating Centers. The electronic clinical chart is used to record data of patients at each visit and periodically revised and updated. All participant subjects signed written informed consent and each center obtained approval by its Ethics Committee.

### Study design

An observational study including HIV infected patients switching to uATV was performed. All patients were 18 years old or more. Patients were included in the study if received uATV from 2000 to 2015. Subjects included in the study were all experienced to antiretroviral therapy. Baseline was defined as the last evaluation before uATV prescription. Patients were followed from baseline (date of the last visit or exam evaluation before uATV prescription) to the time of death, switching from uATV to another regimen, or to the last available visit. The observation was interrupted also when patients were lost to follow up. Patients with any renal, liver, cardiovascular events or non AIDS related cancers before or at switch to uATV were excluded from the present study. A composite end-point (the first occurring of these conditions: liver, cardiovascular, kidney, diabetes, non AIDS related cancer or death events). Renal disease was defined as estimated glomerular filtration rate (eGFR) ≤60 ml/min calculated using Chronic Kidney Disease Epidemiology (CKD-EPI) formula [28], an additional measurement of eGFR within 90 days was acquired to confirm renal impairment. Clinical events of patients were recorded on an electronic clinical chart and reported following a standard classification [11]. Cardiovascular events included diagnosis of acute myocardial infarction, stroke, transient ischemic attack, angina pectoris, coronary bypass, angioplasty, chronic occlusive arterial disease, hypertension. Liver diseases included: hyperbilirubinemia (>2.5 mg/dl), liver fibrosis stage ≥ F3, diagnosis of liver cancer. Diabetes was defined as fasting glucose ≥126 mg/dl or oral anti diabetes therapy or insulin prescription. Discontinuation of uATV was defined as uATV interruption or ATV/r prescription. Causes of uATV discontinuation were categorized as: virological failure, toxicity, simplification, other. A sensitivity analysis limited to patients who reported <4 changes of therapy in the nucleoside/nucleotide (NRTI) backbone (at baseline or in the past) was conducted, in order to exclude potential confounders due to multiple cART switches and multiple virological failures.

### Data collection

Baseline data collected included age, gender, nationality, risk factors for HIV acquisition, HBV and/or HCV co-infections, number of AIDS events, HIV RNA, CD4+ T cell count, ATV/r exposure, cART switches occurred for the backbone. The following parameters were also collected during the follow-up if available: CD4+ T-cell count, HIV RNA, bilirubin levels, γ-glutamyl transferase (γGT) levels, alanine transaminase (ALT) and aspartate transaminase (AST) levels, total and fractioned cholesterol, triglycerides, serum glucose, serum creatinine, platelet count, albumin, blood count, C reactive protein and coagulation markers.

Liver fibrosis estimated by Fib-4 formula [29], eGFR, body mass index (BMI) and systemic inflammation scores (GPS-Glasgow Prognostic Score; mGPS-modified Glasgow Prognostic Score; NLR and PLR) were calculated at baseline and during the follow up using data collected at each time point. Clinical events (AIDS, diabetes, non AIDS related cancers, cardiovascular events, renal and liver diseases), discontinuations of uATV and deaths were recorded at baseline and at each time-point.

### Statistical analysis

Incidence of AIDS and non-AIDS clinical events and of the composite end-point were calculated. Kaplan-Meier analysis was performed to estimate probability of being event-free during follow up. Cox regression analysis, univariate and multivariable analyses (with bi-directional stepwise selection driven by Akaike Information Criterion, AIC), were used to assess predictors of composite clinical end-point. Data collected at baseline and during the follow-up, were input in the models as time updated variables. All analyses were performed using R, the language for statistical computing (https://www.r-project.org/).

## Results

### Baseline characteristics of patients

Four hundred and thirty-six patients were selected (see Table 1). The majority of patients were males (61.5%) and Italians (85.3%). Mean age was 42.7 years (IQR: 37.7–42). The most frequent risk factor for HIV transmission was heterosexual intercourse (47%), followed by injection drug use (25%) and homosexual contact (24%). The rate of positive HCV-Ab was 16.3%. All patients were previously exposed to antiretroviral drugs, including protease inhibitors, before switching to uATV.

**Table 1:** Characteristics of the population at baseline

Variable	N	%
*Qualitative*		
Gender		
Female	168	38.5%
Male	268	61.5%
Risk factors for HIV acquisition		
Heterosexual transmission	203	46.6%
MSM	103	23.6%
IDU	111	25.5%
Other/unknown	19	4.4%
Nationality		
Italian	372	85.3%
Non Italian	64	14.7%
AIDS events		
0	332	76.1%
1	58	13.3%
2	23	5.3%
3+	23	5.3%
CD4+ T cell count (cells/mm^3^)		
≤ 200	70	16.1%
> 200	366	83.9%
HIV RNA (copies/mL)		
≤ 50	269	61.7%
51-5000	72	16.5%
> 5000	95	21.8%
Fib-4 score		
≥ 1.45	353	81%
1.46-3.25	66	15.1%
> 3.25	17	3.9%
HBsAg		
Negative	354	81.2%
Positive	8	1.8%
Unknown	74	17%
HCV Ab		
Negative	256	58.7%
Positive	71	16.3%
Unknown	109	25%
cART exposure		
NRTI	432	99.1%
TDF	254	58.3
ABC	306	70.2%
NNRTI	251	57.6%
PI	436	100%
Other	57	13.1%
*Quantitative*	Median	IQR
Age	42.72	(37.68-47.02)
BMI (Kg/m^2^)	23.64	(20.96-25.93)
CD4+ T cell count cells/mm^3^	486.4	(275.2-660)
Total cholesterol (mg/dl)	191.4	(153-225)
HDL	44.81	(35-51)
LDL	116.5	(90.75-136)
Triglycerides (mg/dl)	183.3	(89-221)
Serum glucose (mg/dl)	88.89	(82-97)
Bilirubin (mg/dl)	0.817	(0.4-1.01)
γGT (IU/L)	63.38	(20-62)
eGFR (mL/min/1.73 m^2^)	101.9	(90.28-114.2)
Number of switches in the NRTI backbone	4	(2-8)

*Abbreviations: N* number, *HIV* human immuno-deficiency virus, *AIDS* acquired immune deficiency syndrome, *MSM* men have sex with men, *IDU* intravenous drug use, *HCV Ab* hepatitis C virus antibodies, *HBsAg* Hepatitis B virus surface antigen, *Fib-4* fibrosis four score, *PI* proteases inhibitor, *cART* combination antiretroviral therapy, *γGT* γ-glutammil-transpeptidase, *BMI* body mass index, *eGFR* estimated glomerular filtration rate, *NNRTI* Non-Nucleoside Reverse Transcriptase Inhibitors, *NRTI* Nucleoside Reverse Transcriptase Inhibitors, *ABC*: abacavir, *IQR* interquartile range

### Clinical outcomes

**Fig. 1:**
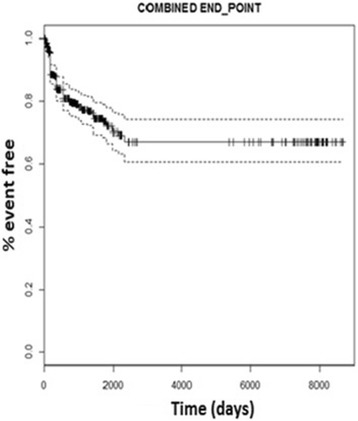
Kaplan-Meier estimation of probability of combined clinical outcome. (*Dashed lines* represent 95% confidence intervals)

Patients were observed for a median time of 882 days (IQR: 252–1,769) under uATV. For the composite clinical end-point, 40 and 30 patients contributed after day 200 and 300, respectively. Ninety three clinical events (3 cardiovascular events, 20 kidney diseases, 33 liver diseases, 9 cancers, 21 diabetes, 7 AIDS events), and 19 deaths occurred during the follow up. Eighty nine composite clinical events were observed (Fig. 1). Causes of death were: AIDS for 6/19 patients, cardiovascular event 1/19, non AIDS related cancer 1/19, other reasons 11/19 patients. An incidence of 3.7 events per 100 person-year of follow-up (PYFU) was estimated. Three hundred eighty-six patients (88.5% of total subject enrolled) discontinued uATV during the follow up. For uATV discontinuations, 48 and 36 patients contributed with data after 2000 and 3000 days, respectively. The cumulative incidence of discontinuations of uATV was 16.6 per 100 PYFU. Forty-seven per cent patients switched to alternative drugs and 53% patients were prescribed ATV/r. Fourteen percent of the patients stopped uATV for toxicity, 19% for simplification, 15% for virological failure, 5% for patient’s choice, and 47% for other/unknown reasons including avoidance of off label prescriptions in patients who did not have any other reasons for stopping uATV.

### Predictors of clinical outcomes

**Fig. 2:**
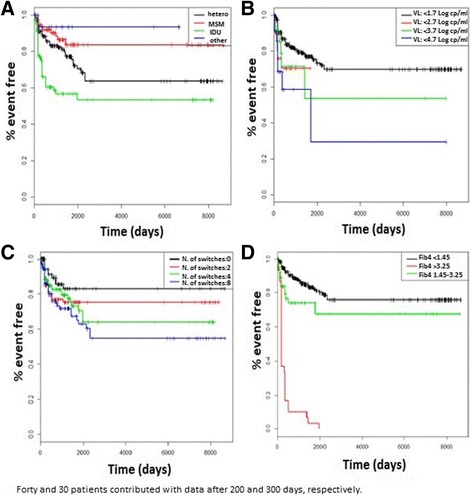
Incidence of the composite outcome by risk factor for HIV acquisition (panel **a**), HIV RNA (panel **b**), number of switches in the nucleoside/nucleotide backbone (panel **c**), and Fib-4 score (panel **d**). List of abbreviations MSM: men have sex with men, IDU: intravenous drug use, VL: HIV RNA viral load, Fib4: fibrosis 4 score

Incidence of the composite end point according to risk factors for HIV acquisition, HIV RNA, Fib-4 score, and number of NRTI switches in the cART backbone are depicted in Fig. 2. At multivariable analysis (Table 2), factors associated with the composite clinical end-point were: intravenous drug use as risk factor for HIV acquisition *vs.* heterosexual intercourses [HR: 2.608, 95%CI 1.31–5.19, *p* = 0.0063], HIV RNA per Log_10_ copies/ml higher [HR: 1.612, 95% CI 1.278–2.034, *p* < 0.0001], switches of cART backbone (performed to compose the uATV regimen under study or occurred in the past) per number of switches higher [HR: 1.085, 95% CI 1.025–1.15, *p* = 0.0051], number of AIDS events prior or at baseline [HR: 1.278, 95% CI 1.072–1.523, *p* = 0.0063], serum glucose levels [HR: 1.034, 95%CI 1.026–1.042, *p* < 0.0001], γGT levels [HR: 1.004, 95%CI 1.003–1.005, *p* < 0.0001], Fib-4 score values per unit higher [HR: 1.030, 95%CI 1.018–1.043, *p* < 0.0001] and NLR score values per unit higher [HR: 1.002, 95%CI 1.001–1.004, *p* = 0.003]. Patients with homo-bisexual intercourses as risk factor for HIV acquisition with respect to heterosexual [HR: 0.379, 95%CI 1.402–5.449, *p* = 0.003], LDL cholesterol [HR: 0.994, 95%CI 0.989-1, *p* = 0.0377] and eGFR [HR: 0.96, 95%CI 0.947–0.972, *p* < 0.0001] values per unit higher showed a significant lower risk of composite clinical outcome. Also, NLR values per Log_10_ higher [HR: 1.179, 95%CI 1.048–1.325, *p* = 0.006] showed statistically significant associations with uATV discontinuation (any causes). When the analysis was performed in a subset of 219 patients who reported <4 cART changes in the NRTI backbone (at baseline or in the past), 35 composite events were observed accounting for a cumulative incidence of 0.3 per 100 PYFU. In this analysis, the association of NLR with the composite clinical outcome was not statistically significant at univariate analysis [HR: 0.999, 95%CI 0.992-1, *p* = 0.79], so it was not included in the multivariable model. At multivariable analysis, factors associated with the composite clinical end-point were higher triglycerides levels [HR: 1.003, 95%CI 1.001-1.004, *p* < 0.0001], higher serum glucose [HR: 1.065, 95%CI 1.040–1.089, *p* < 0.0001] and Fib-4 score per unit higher [HR: 1.108, 95%CI 1.048–1.172, *p* < 0.0001].

**Table 2:** Univariate and multivariable analyses for composite clinical outcome

	Univariate analysis		Multivariable analysis (stepwise AIC)	
*Variable*	HR (95% CI)	*p-*value	HR (95% CI)	*p-*value
Risk factors for HIV acquisition				
MSM *vs.* heterosexual transmission	0.547 (0.28-1.068)	0.0773	0.379 (0.174-0.828)	0.0149
IDU *vs.* heterosexual transmission	2.165 (1.383-3.388)	0.0007	2.608 (1.31-5.19)	0.0063
Log_10_ HIV RNA (copies/mL)	1.516 (1.264-1.818)	<0.0001	1.612 (1.278-2.034)	<0.0001
LDL cholesterol (mg/dl)	0.995 (0.99-1.001)	0.0819	0.994 (0.989-1)	0.0377
Serum glucose (mg/dl)	1.026 (1.02-1.033)	<0.0001	1.034 (1.026-1.042)	<0.0001
γGT (IU/L)	1.003 (1.002-1.004)	<0.0001	1.004 (1.003-1.005)	<0.0001
eGFR (mL/min/1.73 m^2^)	0.968 (0.956-0.98)	<0.0001	0.959 (0.947-0.972)	<0.0001
Fib-4	1.033 (1.022-1.043)	<0.0001	1.03 (1.018-1.043)	<0.0001
Log_10_ NLR	1.31 (1.084-1.582)	0.0052	1.319 (1.047-1.662)	0.0188
Co-infections with hepatitis viruses				
HBsAg unknown *vs*. HBsAg negative	1.067 (0.612-1.86)	0.82	2.525 (1.055-6.043)	0.0374
HCV Ab unknown *vs.* HCV Ab negative	1.495 (0.907-2.466)	0.1148	0.376 (0.164-0.86)	0.0205
Number of switches in the NRTI backbone	1.08 (1.037-1.125)	0.0002	1.085 (1.025-1.15)	0.0051
Number of AIDS events	1.205 (1.089-1.334)	0.0003	1.278 (1.072-1.523)	0.0063

*HR* hazard ratio, *AIC* Akaike Information Criterion, *HIV* human immuno-deficiency virus, *AIDS* acquired immune deficiency syndrome, *MSM* men have sex with men, *IDU* intravenous drug use, *HCV Ab* hepatitis C virus antibodies, *HBsAg* Hepatitis B virus surface antigen, *Fib-4* fibrosis four score, *PI* proteases inhibitor, *cART* combination antiretroviral therapy, *γGT* γ-glutammil-transpeptidase, *eGFR* estimated glomerular filtration rate, *NRTI* Nucleoside/nucleotide reverse transcriptase inhibitors, *CI* confidence interval, *NLR* neutrophil/lymphocytes ratio, *HR* hazard ratio, *Fib-4* fibrosis 4 score

For quantitative variables, *HR* indicates the risk for each unit increase

## Discussion

This study evaluated long-term clinical outcomes of HIV infected patients included in the Italian MASTER cohort after switching to a simplification regimen which included uATV. Findings of the present study are original because, for the first time, we explored the possible association of both inflammation and liver fibrosis scores with clinical events. Moreover, this is the first study which evaluated long-term clinical outcomes in patients treated with uATV.

High levels of inflammation are predictors of clinical events and mortality in HIV infected patients [30, 31]. In particular, studies from our cohort demonstrated that NLR score is an independent predictor of mortality for cancer [25, 32, 33], and cardiovascular events [24]. Moreover, it was demonstrated that may predict death in patients with liver cirrhosis [34]. Herein we confirm that a high NLR is a predictor of clinical events when a composite clinical outcome is used in patients prescribed uATV. Conversely, we did not observe any significant associations between clinical outcome and GPS or PLR. So, probably, these scores (GPS and PLR) are predictors of progression of disease and mortality but only in specific subgroups of HIV infected patients, such those with cancer [25, 32].

The fact that, in a subset of patients with experience of <4 changes in the NRTI backbone, NLR was not significantly associated with the composite clinical outcome may indicate that this score is more reliable in those who were more heavily exposed to cART, with a longer disease history, and with a greater risk of complications. Therefore, our results suggest that NLR warrants more attention and should be measured in HIV infected patients, especially in the most fragile, even when they are treated with “metabolic friendly” regimens (such as cART including uATV).

In the present study, we found that intravenous drug use and higher γGT levels (that may be considered as a proxy for alcohol abuse [35]) are associated with the composite clinical outcome. Also, higher levels of serum glucose were associated with clinical events. These findings are not unexpected because it is well known that life-style factors, such as nutritional habits, alcohol abuse, intravenous drug use or smoking exert an important role for the risk of clinical events even in HIV infected patients [27, 36–39]. So, we confirm that targeted interventions to correct these modifiable risk factors should be prioritized in HIV infected patients to reduce risk of clinical events even when more “metabolic friendly” regimens are prescribed.

The Fib-4 score is a reliable marker of liver fibrosis progression and clinical events in HIV infected patients [26, 27]. Indeed, we also found a statistically significant association of higher Fib-4 scores with the composite clinical outcome. Hence, our results suggest that patients treated with uATV with higher estimated liver fibrosis stage should be considered more at risk for clinical events. This is particularly applied to patients co-infected with HCV, for whom uATV was one of the preferred regimens [7, 8], and to those with higher NLR. Overall, these results reinforce the importance of eradicating HCV as it is the main driver of liver fibrosis progression, to reduce both liver complications and non-AIDS related co-morbidities, especially if Fib-4 and NLR remain elevated.

Results of our study are affected by several limitations. First, high incidence rates of uATV discontinuations (in most cases for “administrative” reasons to reduce off-label prescriptions) may have reduced the length of observation and, therefore, the statistical power of the study may have been limited further. However, the impact of NLR was demonstrated on the risk of both clinical complications and discontinuations of uATV.

Second, our study did not include a control group. Clearly, a control group would be of value, however, we believe that comparison with other cART regimens is not recommended in this study. Indeed, the main inclusion criteria uATV prescription; extending the patients’ selection to other cART types would require an adjustment based on the cART type. The cART effect may be so large to wash out the predictors of clinical events that we are interested in for the subpopulation of uATV. Nonetheless, we recognize the importance of comparing event rates with other regimens. An option could have been to match the uATV population for baseline characteristics. But finding suitable controls is difficult (if not impossible). Indeed, patients prescribed uATV in the past had much different characteristics with respect to patients treated with alternative regimens: in clinical practice Italian physicians tended to prescribe uATV in those with a higher rate or risk of co-morbidities, confections with viral hepatitis or intolerance to ritonavir as a booster. Moreover, it is difficult to set an appropriate baseline for the control group (i.e. patients with similar characteristics should have changed one or more drugs after a comparable time period, treatment initiation, or occurrence of certain adverse event). If the control is not suitable we would have introduced several confounder-by-indication biases.

Third, albeit alternative regimens are preferred over uATV when metabolic or liver complications are of concern, our results (especially the potential impact of persisting inflammatory state estimated through NLR) may be of general interest, provoking further investigations. Since uATV may have a smaller effect on residual HIV RNA which may increase, in its turn, levels of inflammation, it is important conduct further studies in patients receiving different cART regimens.

## Conclusions

This work showed that NLR score is associated with clinical events in patients prescribed uATV, especially if they were intravenous drug users with uncontrolled HIV RNA, higher liver fibrosis stages, high γGT and serum glucose levels, and more heavily pre-treated with cART. In conclusion, the role of NLR in predicting the risk of non AIDS related complications and mortality should be investigated in further studies, particularly in most fragile patients (either for HCV co-infection or metabolic complications) such as those who were usually treated with uATV in clinical practice.

## Abbreviations

ABC: Abacavir
AIC: Akaike information criterion
AIDS: Acquired immune deficiency syndrome
ALT: Alanine transaminase
AST: Aspartate transaminase
ATV: Atazanavir
ATV/r: Atazanavir/ritonavir
BMI: Body mass index
cART: Combination antiretroviral therapy
CI: Confidence interval
eGFR: Estimated glomerular filtration rate
Fib-4: Fibrosis four score
GPS: Glasgow prognostic score
HBsAg: Hepatitis B virus surface antigen
HBV: Hepatitis B virus
HCV: Hepatitis C virus
HIV: Human immuno-deficiency virus
HR: Hazard ratio
IDU: Intravenous drug use
IQR: Interquartile range
MASTER Cohort: Standardized Management of Antiviral Therapy Cohort
mGPS: Modified Glasgow prognostic score
MSM: Men have sex with men
NLR: Neutrophil/lymphocyte ratio
NNRTI: Non-nucleoside reverse transcriptase inhibitors
NRTI: Nucleoside reverse transcriptase inhibitors
PI: Proteases inhibitor
PLR: Platelet/lymphocyte ratio
PYFU: Person-year of follow-up
uATV: Unboosted atazanavir
γGT: γ-glutammil-transpeptidase
